# Mobile Phone-Based Population Flow Data for the COVID-19 Outbreak in Mainland China

**DOI:** 10.34133/2021/9796431

**Published:** 2021-06-18

**Authors:** Xin Lu, Jing Tan, Ziqiang Cao, Yiquan Xiong, Shuo Qin, Tong Wang, Chunrong Liu, Shiyao Huang, Wei Zhang, Laurie B. Marczak, Simon I. Hay, Lehana Thabane, Gordon H. Guyatt, Xin Sun

**Affiliations:** ^1^College of Systems Engineering, National University of Defense Technology, Changsha, China; ^2^Department of Global Public Health, Karolinska Institute, Stockholm, Sweden; ^3^Chinese Evidence-Based Medicine Center, West China Hospital, Sichuan University, Chengdu, China; ^4^Department of Health Research Methods, Evidence and Impact, McMaster University, Hamilton, Canada; ^5^West China Biomedical Big Data Center, West China Hospital, Sichuan University, Chengdu, China; ^6^Department of Health Metrics Sciences, School of Medicine, University of Washington, Seattle, WA, USA

## Abstract

**Background:**

Human migration is one of the driving forces for amplifying localized infectious disease outbreaks into widespread epidemics. During the outbreak of COVID-19 in China, the travels of the population from Wuhan have furthered the spread of the virus as the period coincided with the world's largest population movement to celebrate the Chinese New Year.

**Methods:**

We have collected and made public an anonymous and aggregated mobility dataset extracted from mobile phones at the national level, describing the outflows of population travel from Wuhan. We evaluated the correlation between population movements and the virus spread by the dates when the number of diagnosed cases was documented.

**Results:**

From Jan 1 to Jan 22 of 2020, a total of 20.2 million movements of at-risk population occurred from Wuhan to other regions in China. A large proportion of these movements occurred within Hubei province (84.5%), and a substantial increase of travels was observed even before the beginning of the official Chinese Spring Festival Travel. The outbound flows from Wuhan before the lockdown were found strongly correlated with the number of diagnosed cases in the destination cities (log-transformed).

**Conclusions:**

The regions with the highest volume of receiving at-risk populations were identified. The movements of the at-risk population were strongly associated with the virus spread. These results together with province-by-province reports have been provided to governmental authorities to aid policy decisions at both the state and provincial levels. We believe that the effort in making this data available is extremely important for COVID-19 modelling and prediction.

## 1. Introduction

On December 31, 2019, the National Health Commission of China informed the World Health Organization (WHO) about a cluster of emergent cases with atypical pneumonia in Wuhan [1]. One week later, Chinese scientists isolated a novel coronavirus from the cases [2]. Subsequently, a familial cluster of atypical pneumonia occurred in Shenzhen on January 10, 2020, confirming the human-to-human transmission of the virus [3]. On January 30, 2020, WHO announced that the outbreak of the novel coronavirus pneumonia (COVID-19) in China constitutes a public health emergency of international concern (PHEIC) [4]. Until Dec 17, 2020, it has caused 72,851,747 confirmed cases and 1,643,339 confirmed deaths in over 222 countries, areas, or territories globally [5].

The epidemic center of disease outbreak in China, Wuhan, is the seventh largest city in China with approximately nine million registered residents and two million nonpermanent residential population [6]. Built alongside the Yangtze river and located at the central part of the country, the city has a highly complex transportation network, including air, water, and advanced road and rail systems, which can reach nearly all cities across China within a day [7]. Moreover, the disease outbreak coincided with the national population migration to celebrate the Chinese New Year (i.e., Chinese Spring Festival Travel, starting January 10, 2020) [8]. As the world's largest event of population movement, more than three billion travels by airplanes, high-speed trains, and buses can occur in a highly condensed time period, usually 40 days [8]. The unexpected disease outbreak in Wuhan, compounded with the intensive movements of a large population with access to a diversity of transportation, represents an unprecedented threat of spread of the virus across the nation. As of Jan 22, 2020, a total of 571 patients were diagnosed in 25 provinces of mainland China, and four countries reported cases [9]. Wuhan announced an immediate lockdown policy on Jan 23, 2020, when all public transport, including buses, railways, flights, and ferry services were suspended [10].

In managing the disease outbreak, previous work usually relied on very early data (e.g., small number of cases) to develop predictions about the potential number of cases [11]. However, such predictions are premature and do not address the extent to which the virus spread results from population movements and other demographic and environmental factors. We believe that the population movement is the main mechanism for the virus spread at this stage, in which a large number of infected individuals would have travelled with no symptoms (during the incubation period). Human mobility has long been integrated in epidemiological models for predicting the development of infectious diseases [12]. However, due to the lack of accurate and representative population-level data, many studies often rely on hypothesized statistical models, e.g., gravity models [13, 14], and radiation models [15], to generate estimates about population movements by assuming a functional relationship between the likelihood of movement and other factors such as distance and population size. On the other hand, large-scale movement data has been extracted from transportation systems [11], mobile devices [16, 17], and geo-tagged online posts [18, 19]. Among those, mobile phone data has been recognized as the most accurate and reliable source [20] and has been used in a variety of responses to diseases [21–25].

As the present of this work, a number of studies on how population flow, measured by flight data [11], internet data with location-based services (LBS) [19, 26–28], etc., has contributed the spreading of COVID-19 have been reported. However, the validity and representativeness of such data is largely unknown due to restrictions of access and lack of comparison with ground truth. For example, the mobility trends offered by Apple and Google, for which the movement of people is extracted for those with an Apple or Android device using mapping apps, cannot cover users who do not own a smart phone or places where access to their services is limited [29–31]. A few other mobile phone-based mobility datasets which were made public for the response of COVID-19 were either extracted from small samples of users [32] or aggregated on a short period such that the temporal variation of mobility cannot be evaluated [33]. In an effort to fill in this gap of knowledge, in this study, we make available an anonymous and aggregated mobility dataset extracted from mobile phones at the national level, describing the outflows of population travelled from Wuhan during the first month of the outbreak, containing the periods before and after city lockdown. The data was extracted on a daily basis for the purpose of decision making on containing the spreading, and it includes the date and destination city, as well as the amount of population flow during the whole January of 2020. We have also collected the case reporting data on the daily number of confirmed cases until March 15, 2020 to show that the spreading of COVID-19 in China is primarily driven by population flow from Wuhan. We believe that the effort in making this data available to all researchers is extremely important for COVID-19 modelling and prediction.

## 2. Materials and Methods

### 2.1. Experimental and Technical Design

**Figure 1:**
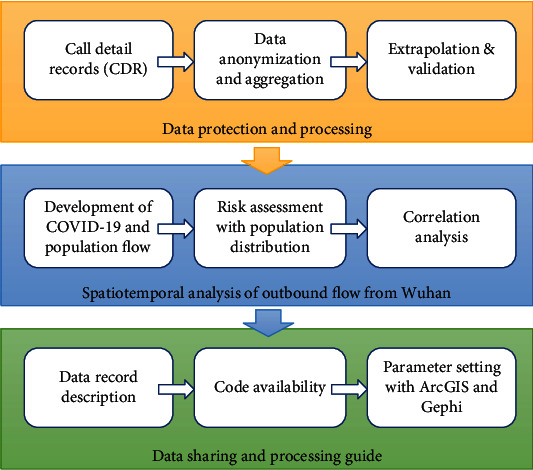
Flowchart of data processing and spatiotemporal analysis.

In this study, we present a complete dataset, describing the population outbound flow from Wuhan during the outbreak of COVID-19 in 2020. The mobility data was extracted from call detail records (CDR) of all users from one of the largest operator, China Unicom, and was extrapolated to the whole network to build movement estimates for those who have left Wuhan to other regions of China, spreading infection risks across the country. The general framework of this paper is described in Figure 1. First, the data was processed anonymously and then aggregated into cross-city/prefecture-level mobility matrices on a daily basis. The number of movements between cities was then extrapolated to the whole network based on a variety of user demographics and operator coverage differences. Second, we present the general development of COVID-19, together with the change of population flow during the outbreak. The spreading risk was assessed through a 3-D visualization of population distribution of those who have left Wuhan before the lockdown. Lastly, we provide details of the data records and made the data, as well as the codes and parameter settings, available to all researchers to help further studies for COVID-19 modelling and prediction.

### 2.2. Mobile Phone Data and Travels of At-Risk Population

Using national aggregated mobile operator data, we measured the movements of the at-risk population from Wuhan to any other location in mainland China from January 1 to 31, 2020. During this period, an at-risk individual may have travelled multiple times from Wuhan or have visited Wuhan but then back to the residence location. The destination of a movement was defined on a daily basis, and multiple destinations were documented. The data were filtered for individuals who stayed in Wuhan for more than two hours, to exclude the large volume of travelers who merely transit through Wuhan (less than two hours). Daily numbers of users for those who stayed in Wuhan and travelled outside and for those who visited Wuhan but travelled back afterwards were summed to construct a national-level outbound flow matrix, i.e., number of people who migrated from Wuhan to other places of mainland China at the resolution of provincial and district (county) levels. The operator, China Unicom, has helped with extrapolating the data to all users from the whole network; the estimation on the movements of all users is then used for the analysis in this study. Details of the data extrapolation validity and accuracy check are provided in Supporting Information S1.

Data processing and aggregation followed the laws of “Provisions on Protection of Personal Information of Telecommunications and Internet Users (Mainland China)” [34] and with reference to the GSMA (Global System for Mobile Communications Association) guidelines on the protection of privacy in the use of mobile phone data for responding to the Ebola outbreak [35]. The authors had access only to exported and aggregated data at the city level. All mobility data were provided by the operator for emergency responses and were anonymized and aggregated. No personally identifying information was processed in the analysis of this study. It is worth noting that, as all data is processed anonymized and aggregately, it is not possible for the authors to identify or filter users of certain groups; the population flow presented in this article thus provides a representative overview of the general population and cannot be analysed for minority groups.

### 2.3. COVID-19 Case Reporting Data

We measured the spread of the virus from officially reported diagnosed cases. The official reporting of diagnosed cases commenced on January 10, 2020, when the complete genome sequencing was announced, laying the foundation for accurate diagnosis. On the same day, the Chinese Spring Festival Travel began [8]. We collected the official numbers of diagnosed patients with COVID-19 from the National Health Commission of China [36], Chinese Center for Disease Control and Prevention [37], and Health Commission of each province.

### 2.4. Ethical Approval

This study was approved by the Ethics Review Board of West China Hospital, Sichuan University (2020-99).

### 2.5. Statistical Analysis

By developing an aggregated mobility matrix of the estimated number of mobile phone users, we calculated movements of at-risk population from Wuhan to destinations for a division of 2,552 districts (counties) among mainland China. We used a thermodynamic chart of flows at the district (county) level across the country to depict the distribution of outbound movements within and outside Hubei province.

We calculated Pearson's correlation coefficients to measure the association between the number of movements (log-transformation to achieve normal distribution) and number of diagnosed cases (log-transformation), both at the provincial and municipal levels. We reported the correlation coefficients by length of observation (i.e., from January 23 until February 5, 2020). We also reported the coefficients by different time periods for population movements (i.e., the first, second, and third weeks between January 1 and January 22) to test for the difference in the coefficients by mobility patterns.

Statistical analyses were conducted with Stata (V.13.0, StataCorp LP), MATLAB (R2018a, The MathWorks, Inc.), Python (V.3.7.4, Python Software Foundation), and ArcGIS (V.10.2, Esri). The level of statistical significance was set at alpha = 0.05.

## 3. Results

### 3.1. Spread of COVID-19 across China at the Early Stage of Disease Outbreak

**Figure 2:**
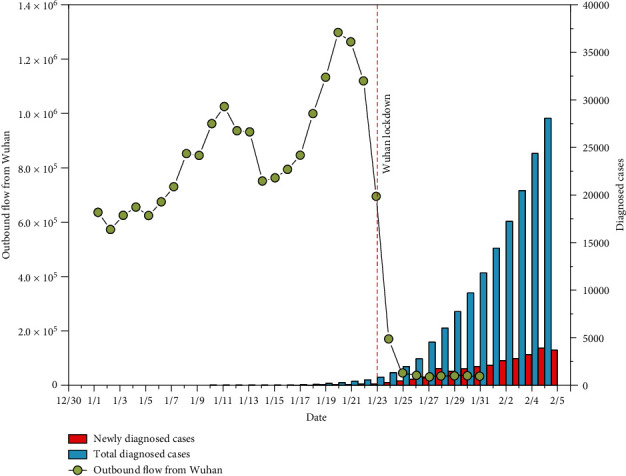
Total outbound flows from Wuhan and number of diagnosed cases.

**Figure 3:**
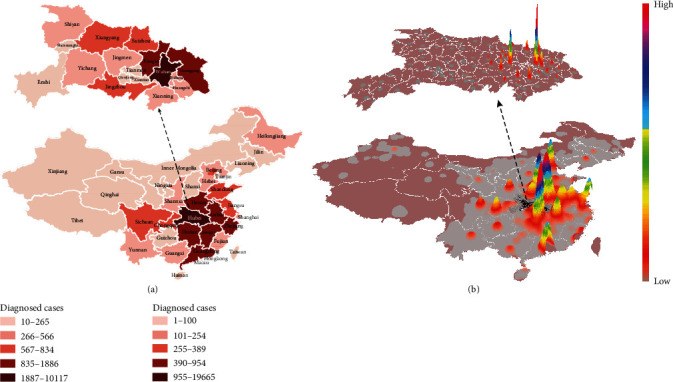
Spatial distribution of the number of diagnosed cases and thermodynamic chart of outbound flows from Wuhan to destinations at the district (county) level across mainland China. (a) Distribution of diagnosed cases in each province, with cities in Hubei province highlighted above; (b) volume of outbound flows from Wuhan between January 1 and 22, 2020. Hubei province is presented at the top of the figure.

A total of 41 cases were firstly confirmed on January 11. The number of diagnosed cases then increased sharply starting January 16 (Figure 2). By February 5, a total of 28,017 cases were diagnosed, and the distribution of diagnosed cases varied substantially across China (Figure 3(a)). Hubei province reported the largest number of cases (19,665). Two adjacent provinces (Henan and Hunan, total 1,562 cases) and the two provinces receiving large population movements (Zhejiang and Guangdong, total 1,898 cases) had more cases than the rest of the provinces.

### 3.2. Mobility Pattern of Population Flow from Wuhan

From January 1 to January 22, a total of 20.2 million movements occurred from Wuhan to other regions, with the smallest number on January 2 (0.57 million travels) and the largest number on January 20 (1.3 million travels, Figure 2). A large proportion of these movements occurred within Hubei province (84.5%). In general, the number of travels from Wuhan increased substantially even before the beginning of the official Chinese Spring Festival Travel. The first peak coincided with the winter break for schools and colleges, as Wuhan hosts the third largest number (1.1 million) of graduate and postgraduate students [38]. The second peak occurred four days before the Chinese New Year Eve (i.e., January 24, 2020). Significant differences were present in the distribution of travel destinations across the country, and the distribution seemed clustered (Figure 3). Provinces neighbouring to Hubei, e.g., Hebei and Hunan, were home to a large proportion of movements. Large cities, including Beijing, Shanghai, and Guangzhou, also received a large number of movements from Wuhan. The mass population flow was halted by the lockdown of Wuhan: on January 23, there were only 62% outbound flow from Wuhan comparing to the preceding day, and the recorded outflow drops rapidly after January 24, with the majority of flow (96%) occurring within Hubei province.

**Figure 4:**
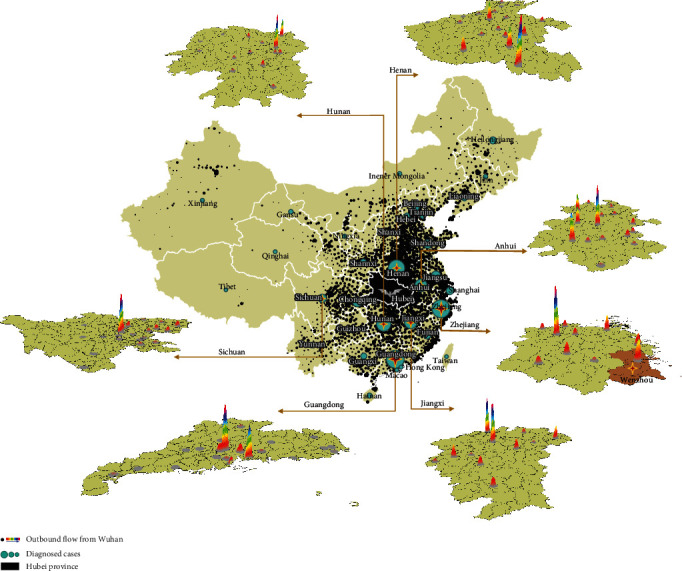
Distribution of outbound flows and diagnosed cases for selected provinces: Henan, Hunan, Anhui, Zhejiang, Guangdong, Jiangxi, and Sichuan, Beijing, Shanghai, and Chongqing. Hubei province is filled with black in the middle of the map. The black spots in other provinces represent the movements at the district level; the green circle represents the number of diagnosed cases; the thermodynamic chart of nine provinces represents the distribution of outbound movements in these provinces.

We also characterized the within-province distribution for movements of at-risk populations from Wuhan in seven representative provinces that had a relatively large number of diagnosed cases, as well as Beijing, Shanghai, and Chongqing (Figure 4). There were notable differences in the spatial distribution of outbound flow between provinces. The destination of movements centered in one or two major cities (usually the largest cities) in some provinces, e.g., Hunan, Guangdong, and Sichuan; the distribution was more homogenous for other provinces including Anhui and Henan.

### 3.3. Time-Dependent Correlation between Population Movements and the Virus Spread

**Figure 5:**
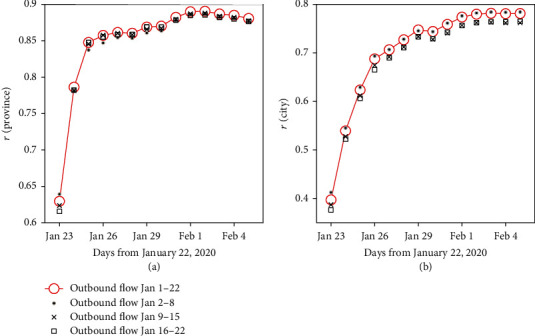
Strength of correlation between population movements and number of diagnosed cases by the length of observation days. (a) All provinces in mainland China excluding Hubei; (b) all cities in mainland China excluding cities in Hubei. Outbound flows are counted for three periods: all days before January 22, January 2 to January 8 (first week), January 9 to January 15 (second week), and January 16 to January 22 (third week).

Figure 5 presented the correlation coefficients between outbound flows from Wuhan before the lockdown and the number of diagnosed cases by the length of observation (i.e., 1 to 14 days after the end of the studied population movements). The strength of association increased over time. At the provincial level, Pearson's correlation coefficients were 0.86 (0.73-0.93, *p* < 0.001) on January 25 and increased up to 0.89 (0.78-0.95, *p* < 0.001) on February 1. The observed pattern was similar at the city level: for all cities in China outside Hubei, the coefficients were 0.63 (0.56-0.69, *p* < 0.001) on January 25 and 0.78 (0.73-0.82, *p* < 0.001) on February 1. The correlations peaked at day 10 (0.89, 0.78-0.95) and day 12 (0.78, 0.74-0.82) for the provincial and city levels, respectively.

### 3.4. Data Sharing and Code Availability

The datasets are provided in Supporting Information S2 and S3, including the mobility dataset and COVID-19 case dataset in CSV format. The mobility dataset included daily individual movements from Wuhan to other regions in mainland China between January 1 and January 31, 2020. The COVID-19 case dataset included the daily number of diagnosed COVID-19 cases in mainland China at the city level between January 10 and March 15, 2020.

Variables in the mobility dataset are shown below:

- *IDcode*: unique identifier for regions, which represented the administrative division code for cities in China
- *Date*: daily date between January 1 and January 31, 2020, which was recorded as YYYYMMDD

When the number of individual movements from Wuhan to a specific region is zero, the date was not displayed.

(iii) *ProvinceName*: name of the province in which the individual movements from Wuhan to other regions, including all 32 provinces in mainland China
(iv) *CityName*: name of the city in which the individual movements from Wuhan to other regions, including 346 cities in mainland China
(v) *Longitude*: the longitude of the specific city (denoted as “point” in “geo resolution”)
(vi) *Latitude*: the latitude of the specific city (denoted as “point” in “geo resolution”)
(vii) *FlowCount*: the number of individual movements from Wuhan to a specific city.

Variables in the COVID-19 cases dataset is shown below:

- *IDcode*: unique identifier for regions, which represented the administrative division code for cities in China
- *ProvinceName*: name of the province in which the COVID-19 case is reported
- *CityName*: name of the city in which the COVID-19 case is reported, including 347 cities in mainland China
- *Population*: population in tens of thousands for each city. The data was manually retrieved from the latest statistical yearbook of each city
- *10-Jan to 15-Mar*: daily cumulative number of diagnosed COVID-19 cases from January 10 to March 15, 2020.

All codes used to clean the data as well as parameter settings used to plot the main figures are provided in Supporting Information S1.

## 4. Conclusion and Discussion

In the past two decades, the world has experienced several virulent disease outbreaks, such as SARS, MERS, Zika, and Ebola [39–44]. Increasing population size and density, complex transportation networks, advanced industrialization, and urbanization initiatives, all have contributed to the rapid and extensive spread of viral outbreaks. These factors are all present as complications in the control of COVID-19 spread. Understanding the travel profiles of at-risk populations, with subsequent implementation of interventions, may serve a critical role in the control of virus spread, especially in the early stages of an outbreak. Using national aggregated mobile operator data, we evaluated movements of the at-risk population from Wuhan and characterized the outbound flow pattern of this at-risk population. In particular, we found that, in the first few weeks of the outbreak, most of the destinations of at-risk populations from Wuhan were either local cities adjacent to Wuhan, provinces adjacent to Hubei province (e.g., Henan, Anhui, Hunan, and Chongqing), or economically developed regions (e.g., Beijing, Shanghai, and Guangdong) (Figure 3). Some provinces had adopted stringent policies intended to block the spread of the virus, such as quarantines and close monitoring of at-risk populations [45, 46].

Another important finding from our study is that two distinct patterns of outbound flows occurred among regions. In some provinces, such as Guangdong and Sichuan, most of the outbound flows were towards the economic center within that region (e.g., Chengdu, Shenzhen, and Guangzhou). In other provinces, such as Henan and Anhui, the travels of at-risk populations were more balanced across the regions. These distinct mobility patterns may also warrant the implementation of differential control strategies. Provinces with a centralized model of movements may need more resources allocated to the most affected city, while those with a distributed model may consider balanced efforts across the regions. These results together with province-by-province reports have been provided to governmental authorities to aid policy decisions at both the state and provincial levels during the initial emergency response period.

The use of national mobility data provides a novel and effective means of supporting the control of infectious disease outbreaks. For future investigation of the early outbreak and understanding of how the spreading of COVID-19 was driven by the mass population flow, we believe that the effort in making this data available is extremely important for researchers in this community. Some study limitations should also be noted. Reporting errors and delays are likely to have occurred in the case data and should be interpreted as reflecting relative rather than absolute differences. Accuracy of estimates may be improved, and effectiveness of quarantines may be assessed when more accurate data are available. In addition, higher orders of mobility matrix [25] (i.e., not only Wuhan to other places, but the all-pair movements and higher order trajectories) could be integrated to support more effective and accurate prevention of the epidemic.

## Data Availability

The data used in this study, including the mobility dataset and COVID-19 case dataset in CSV format, were provided in Supporting Information S2 and S3.

## Supplementary Materials

Supplementary 1 Supporting Information S1: this file contains the following content: (1) details on the mobile phone data extrapolation process and the validation check for data representativeness by comparing mobile phone estimates with population statistics and Baidu LBS data; (2) Python code for preparing outbound flow data for Figures 1 and 2; (3) parameter setting for 3-D visualization of the maps with ArcGIS; (4) parameter setting for node size and layout of Figure 4 with Gephi.

Supplementary 2 Supporting Information S2: this file contains the mobility dataset of daily individual movements from Wuhan to other regions in mainland China between January 1 and January 31, 2020. The data items are introduced in Data Sharing and Code Availability.

Supplementary 3 Supporting Information S3: this file contains the daily number of diagnosed COVID-19 cases, as well as the latest population statistics in mainland China at the city level between January 10 and March 15, 2020. The data items are introduced in Data Sharing and Code Availability.
