# Supplementary material for: Mobile Phone-Based Population Flow Data for the COVID-19 Outbreak in Mainland China
Source: Health Data Sci. 2021 Jun 5;2021:9796431. doi: 10.34133/2021/9796431 (PMC9629681; doi:10.34133/2021/9796431)
Supplement: Supplementary 1 — Supporting Information S1: this file contains the following content: (1) details on the mobile phone data extrapolation process and the validation check for data representativeness by comparing mobile phone estimates with population statistics and Baidu LBS data; (2) Python code for preparing outbound flow data for Figures 1 and 2; (3) parameter setting for 3-D visualization of the maps with ArcGIS; (4) parameter setting for node size and layout of Figure 4 with Gephi. [file 9796431.f1.docx]

**Supporting Information -1**

Data Validation, Processing Code and Parameter Setting

**Title**

*Mobile phone-based population flow data for travels from Wuhan during the COVID-19 outbreak in Mainland China*

Xin Lu^1,2✝*^, Jing Tan^3,4✝^, Ziqiang Cao^1✝^, Yiquan Xiong^3✝^, Shuo Qin^1^, Tong Wang^1^, Chunrong Liu^3^, Shiyao Huang^3^, Wei Zhang^5^, Laurie B. Marczak^6^, Simon I. Hay^6^, Lehana Thabane^4^, Gordon H. Guyatt^4^, Xin Sun^3*^

1. College of Systems Engineering, National University of Defense Technology, Changsha, China; 2. Department of Global Public Health, Karolinska Institute, Stockholm, Sweden; 3. Chinese Evidence-based Medicine Center, West China Hospital, Sichuan University, Chengdu, China; 4. Department of Health Research Methods, Evidence and Impact, McMaster University, Hamilton, Canada; 5. West China Biomedical Big Data Center, West China Hospital, Sichuan University, Chengdu, China; 6. Department of Health Metrics Sciences, School of Medicine, University of Washington, Seattle, WA, USA

^✝^These authors have equal contribution to the article.

^*^corresponding author(s): Xin Lu (lvxin@nudt.edu.cn); Xin Sun (sunxin@wchscu.cn)

**Contents of this supporting information:**

[**S1. Data validation** 3](#_Toc59177604)

[**S1.1 Data extrapolation and validation** 3](#_Toc59177605)

[**S1.2 Estimated coverage ratio** 4](#_Toc59177606)

[**S1.3 Data representativeness** 4](#_Toc59177607)

[*S1.3.1 Comparison of mobile phone estimates with population statistics* 4](#_Toc59177608)

[*S1.3.2 Comparison of mobile phone estimates with Baidu LBS data* 5](#_Toc59177609)

[**S2. Python Code for Data Processing** 7](#_Toc59177610)

[**S2.1 Prepare outbound flow data for Fig.1** 7](#_Toc59177611)

[**S2.2 Prepare outbound flow data for 3-D visualization in Fig.2** 7](#_Toc59177612)

[**S3. Parameter Setting in ArcGIS** 7](#_Toc59177613)

[**S3.1 Anhui Province** 7](#_Toc59177614)

[**S3.2 Sichuan Province** 8](#_Toc59177615)

[**S3.3 Zhejiang Province** 9](#_Toc59177616)

[**S3.4 Jiangxi Province** 9](#_Toc59177617)

[**S3.5 Guangdong Province** 10](#_Toc59177618)

[**S3.6 Hunan Province** 11](#_Toc59177619)

[**S3.7 Henan Province** 11](#_Toc59177620)

[**S4. Parameter Setting in Gephi** 12](#_Toc59177621)

**S1. Data validation**

**S1.1 Data extrapolation and validation**

China Unicom had 318 million active users by the end of 2019^1^, about one quarter of all active mobile phone users in China (the other two operators are China Mobile and China Telecom). In order to enhance the extrapolation and representation of population, user coverage, ratio of calls with other operators, as well as a variety of parameters extracted from the structure of users’ age, gender, etc., were combined and modeled using a machine learning approach by the operator, generating estimates on the number of migrating users from the whole network.

In Figure S1, the flowchart illustrates the process of the extrapolation: first, China Unicom’s users’ locations are extracted as baseline for spatial aggregation; second, phone calls, messaging and other types of communications between China Unicom and other operators’ users are weighted to estimate the number of users in the same district; third, district level user coverage ratios are then summarized for each province; lastly, the real user coverage ratios (market share), which are available at provincial level, are then used to train and obtain the optimize weighting at different districts in step two. This approach is validated with real coverage ratios in four cities, as well as all provinces in China, as shown in Figure S2.


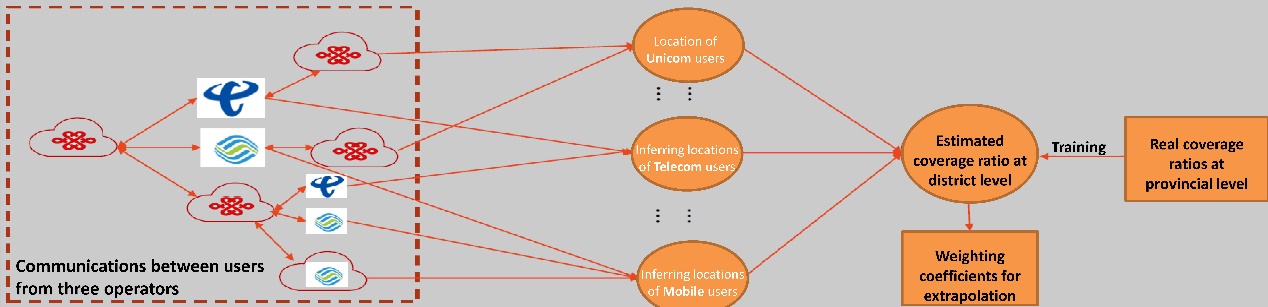


**Figure S1** Schematic Chart for the workflow in data extrapolation.

**Figure S2** Validation of data extrapolation. (A) Comparison of real and estimated user coverage ratio in four selected cities: Hangzhou, Wuhan, Guangzhou, Shenzhen; (B) Comparison of real and estimated user coverage ratio in all provinces (data provided by the operator).

**S1.2 Estimated coverage ratio**

In addition, our analysis indicates that there is a high agreement on the two numbers (i.e., number of users from the whole network and from the operator). And the ratio from the operator’s own sum ($m$) to the network estimates ($m^{*})$, $\theta=m/m^{*}$, varies from county to county. The correlation coefficients of $m$ and $m^{*}$ are all close to one at three admin levels: r=0.9999 for county level, and r=1.0 for both district and provincial level.


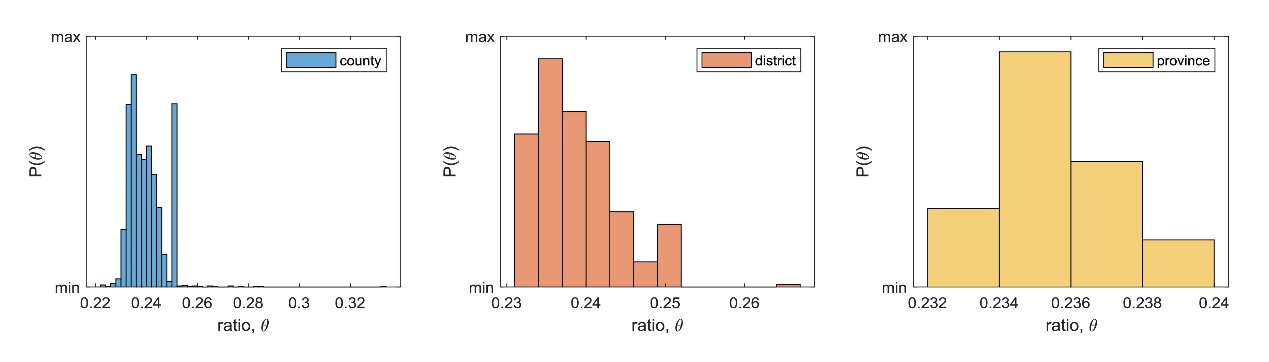


**Figure S3** Ratio of estimated number of users from the whole network, to the number of users from the operator. (a) county level; (b) district level; (c) provincial level.

**S1.3 Data representativeness**

*S1.3.1 Comparison of mobile phone estimates with population statistics*

The advantages of mobile phone data include extensive coverage and high representativeness. For China, the number of mobile phone subscriptions reached 1.6 billion by November 2019, with individuals owning more than one mobile phone number on average^[[1]](#footnote-1)^. A total of 847 million Chinese people used mobile phones to surf the internet, accounting for 99.1 percent of the total netizens.

The penetration rate of mobile phone usage among the population aged 15 to 65 years is almost 100%, estimation to the general population can then be made by extrapolating from the number of users from the whole network against the dependency ratio – the ratio of population ages 0 to 14 and 65+ to those in the labor force (ages 15 to 64). According to the National Bureau of Statistics^[[2]](#footnote-2)^, China has a youth dependency ratio of 23.7% and an old-age dependency ratio of 16.8%.

We used data provided by China Unicom to evaluate the consistency between population estimated from mobile phone data with the above adjustments, and the official statistics on residential population in Shanghai at district level (Figure S4). For each district in Shanghai, the estimated number of residents agrees well with the official statistics, with R^2^=0.99.

**Figure S4** Consistency of mobile phone users’ estimates with the population data at district level in Shanghai. Data provided by China Unicom.

*S1.3.2 Comparison of mobile phone estimates with Baidu LBS data*

To investigate the difference between mobile phone data estimates and the flow generated by one of the most widely used open source, Baidu LBS (location-based services),^[[3]](#footnote-3)^ we compared the top 100 destination cities from both datasets, as presented in Figure S5. Generally, the cities estimated with heavy amount of flows from Wuhan by Baidu LBS, were also top destination cities estimated by the mobile phone data. There is a highly consistency for the first 20 to 30 cities outside Wuhan, and the overall spearman correlation is *r*=0.93 (0.90 to 0.95, *p*<0.001).

**Figure S5** Comparison on the order of top 100 destination cities from Wuhan on January 22, 2020, based on flows estimated from China Unicom and Baidu.

**S2. Python Code for Data Processing**

**S2.1 Prepare outbound flow data for Fig.1**

1. # prepare outbound flow data for Fig.1
2. **import** pandas as pd
3. # read the Mobile phone data
4. df = pd.read_csv("SI-1 Human mobility.csv")
5. # calculate the total outbound flows from Wuhan from January 1 to 31, 2020
6. df.groupby("Date").sum().to_csv("the total outbound flows.csv")

**S2.2 Prepare outbound flow data for 3-D visualization in Fig.2**

1. # prepare outbound flow data for 3-D visualization in Fig.2
2. **import** pandas as pd
3. df = pd.read_csv("SI-1 Human mobility.csv")
4. # select the population flows from Wuhan from January 1st to January 22th in mainland China
5. df1=df[~df['Date'].isin(["20200123","20200124","20200125","20200126","20200127","20200128","20200129","20200130","20200131"])]
6. #The total outbound from Wuhan
7. df2 = df1.groupby(["ProvinceName","CityName",],as_index=False)["FlowCount"].sum()
9. #save the data
10. df2.to_csv("the outboundflow from Wuhan from January 1st to January 22th.csv")

**S3. Parameter Setting in ArcGIS**

**S3.1 Anhui Province**


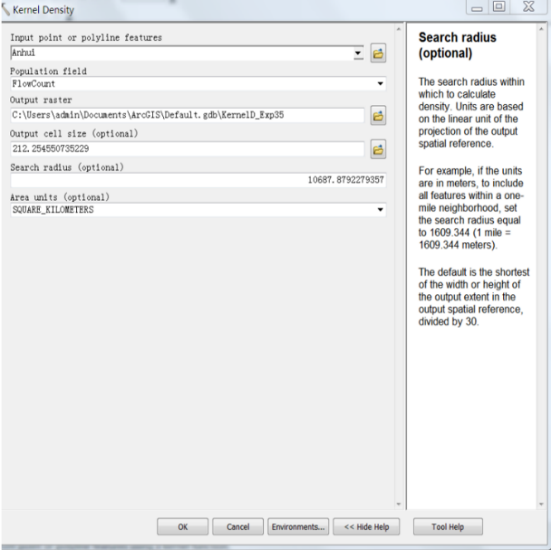

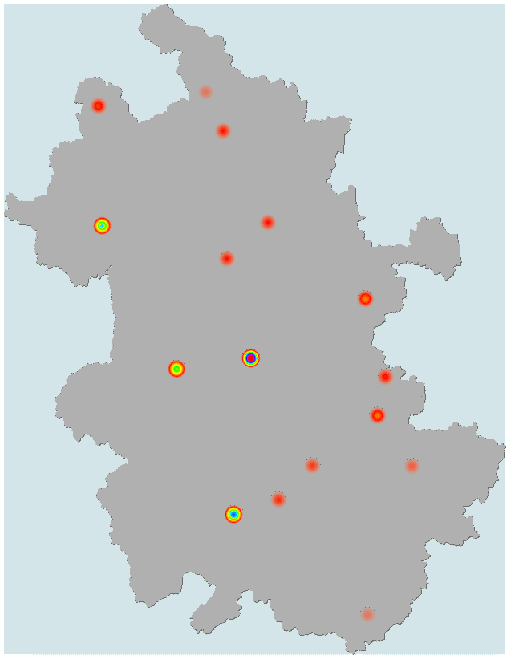


**Figure S6** Parameter setting for Anhui in ArcMap


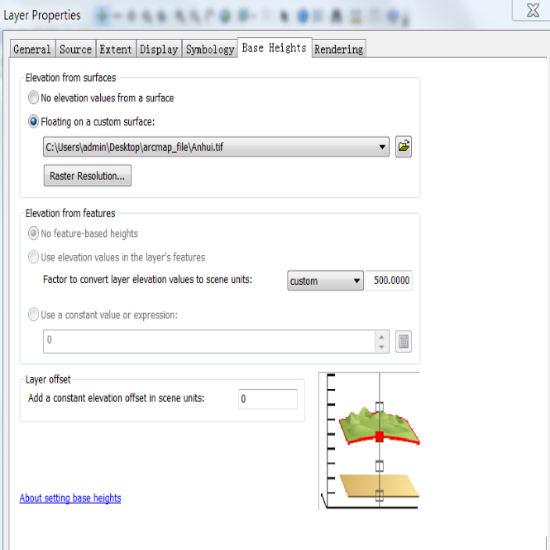

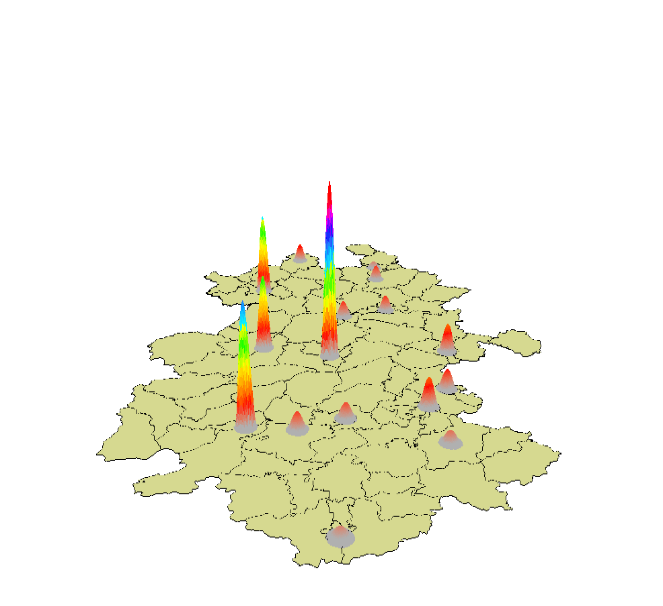


**Figure S7** Parameter setting for Anhui in ArcScene

**S3.2 Sichuan Province**


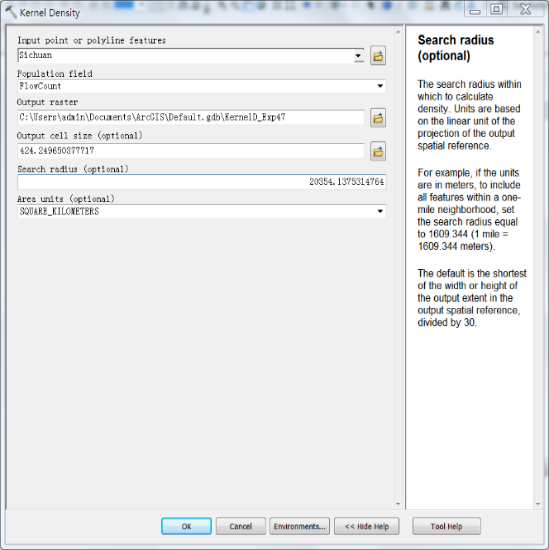

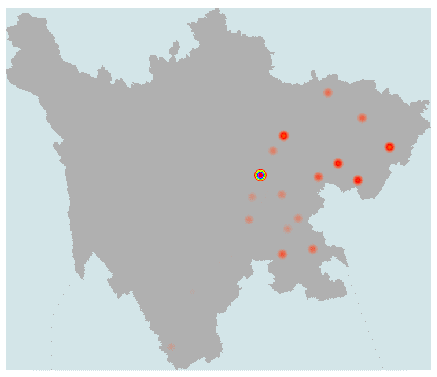


**Figure S8** Parameter setting for Sichuan in ArcMap


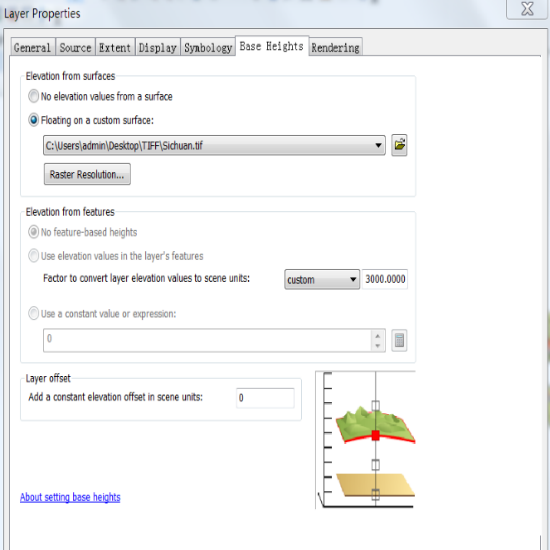

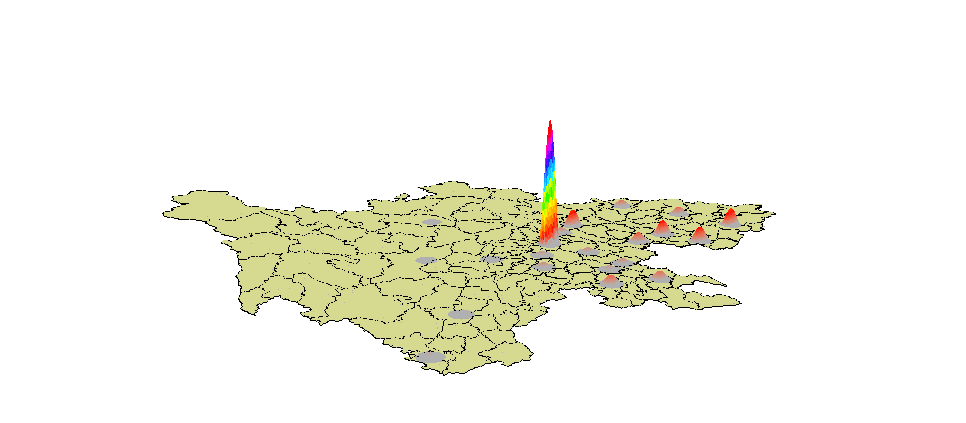


**Figure S9** Parameter setting for Sichuan in ArcScene

**S3.3 Zhejiang Province**


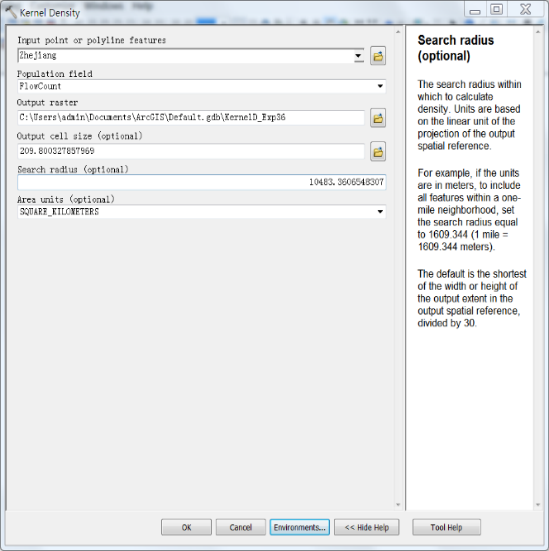

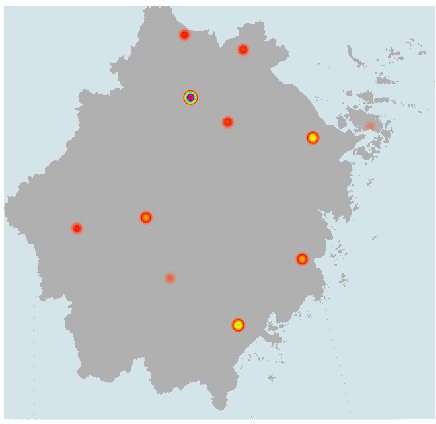


**Figure S10** Parameter setting for Zhejiang in ArcMap


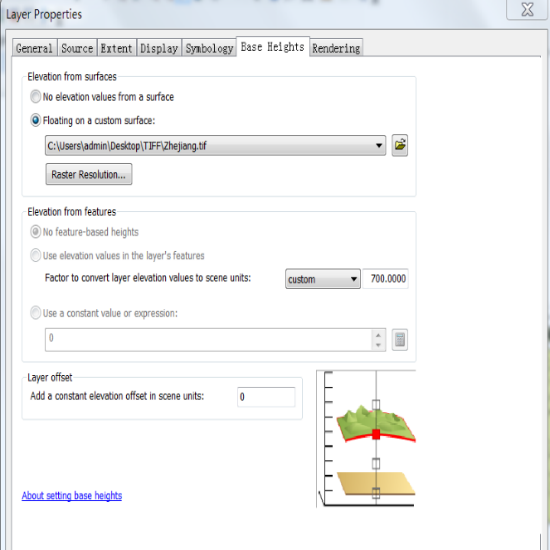

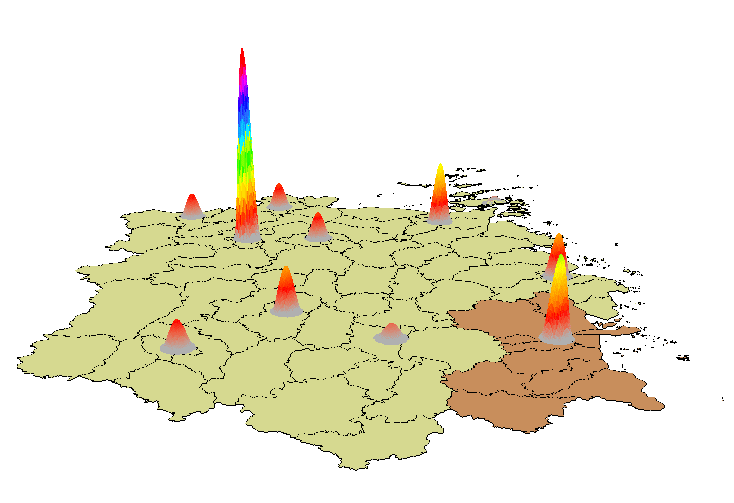


**Figure S11** Parameter setting for Zhejiang in ArcScene

**S3.4 Jiangxi Province**


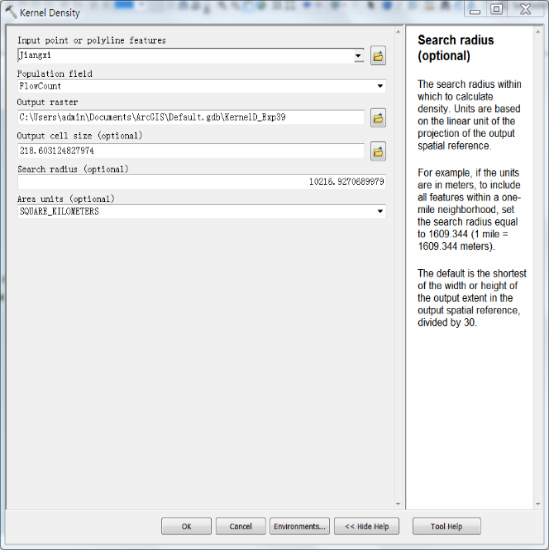

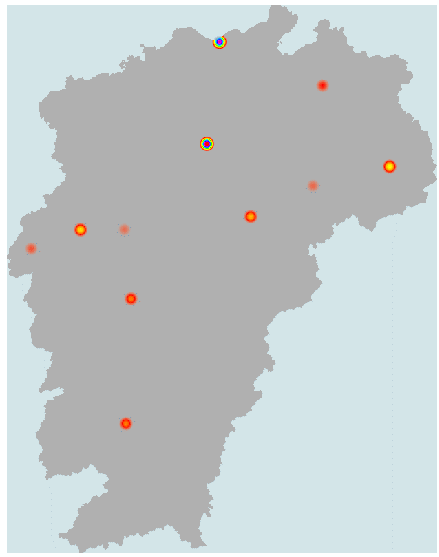


**Figure S12** Parameter setting for Jiangxi in ArcMap


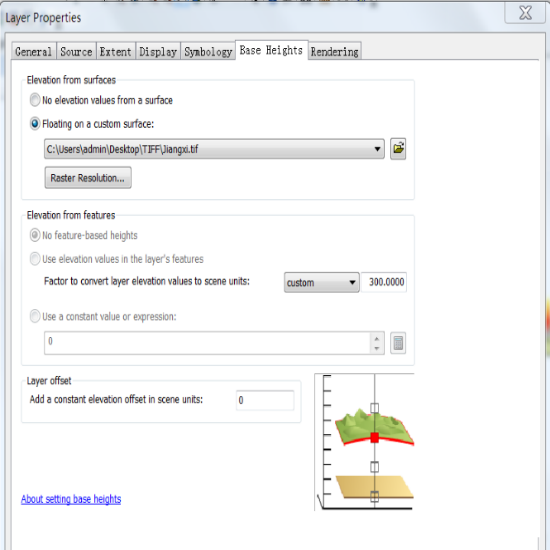

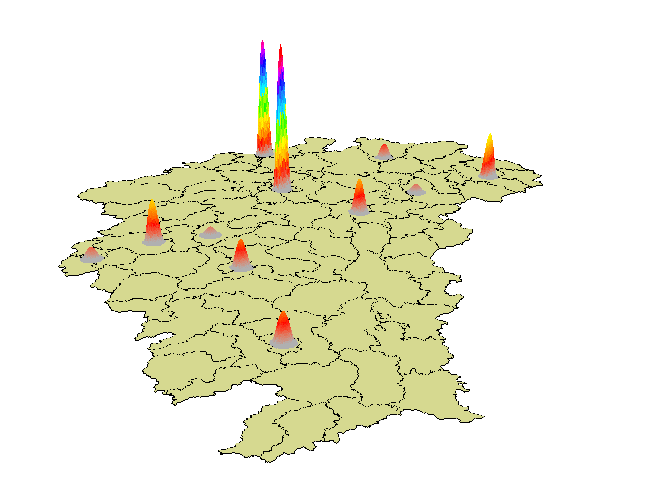


**Figure S13** Parameter setting for Jiangxi in ArcScene

**S3.5 Guangdong Province**


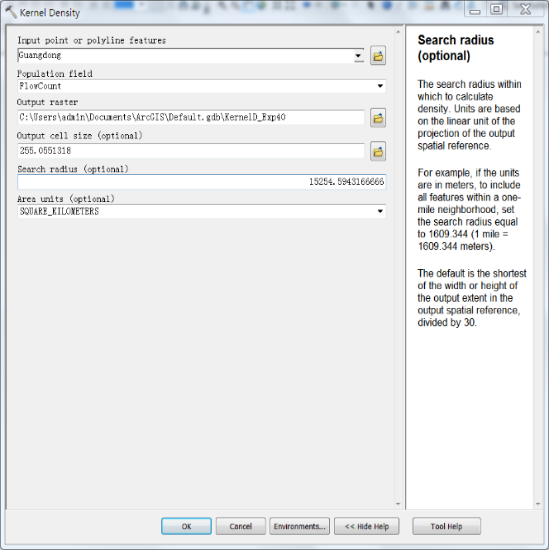

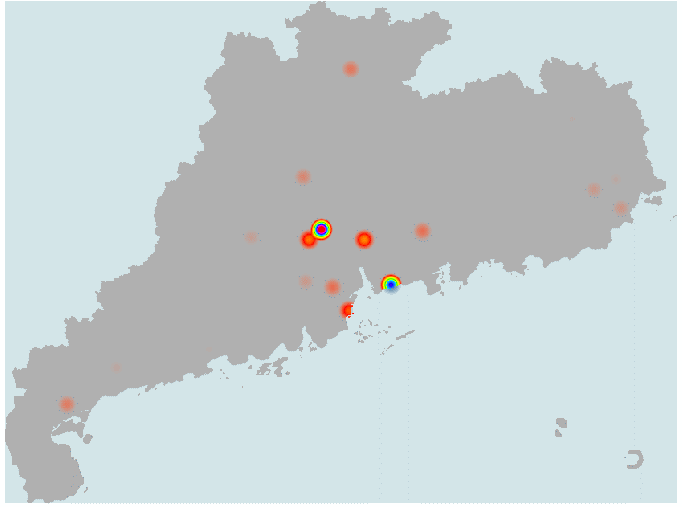


**Figure S14** Parameter setting for Guangdong in ArcMap


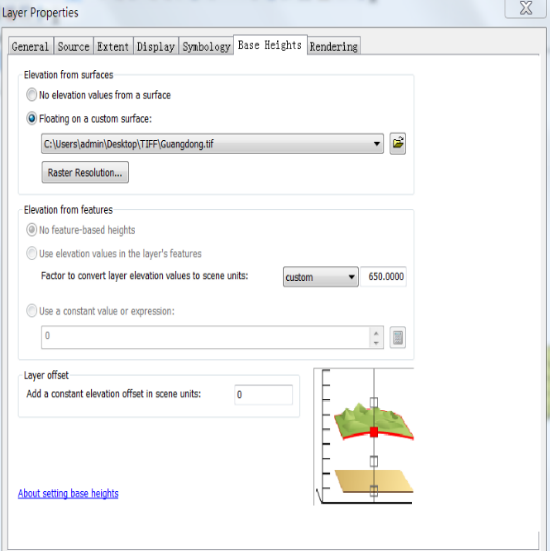

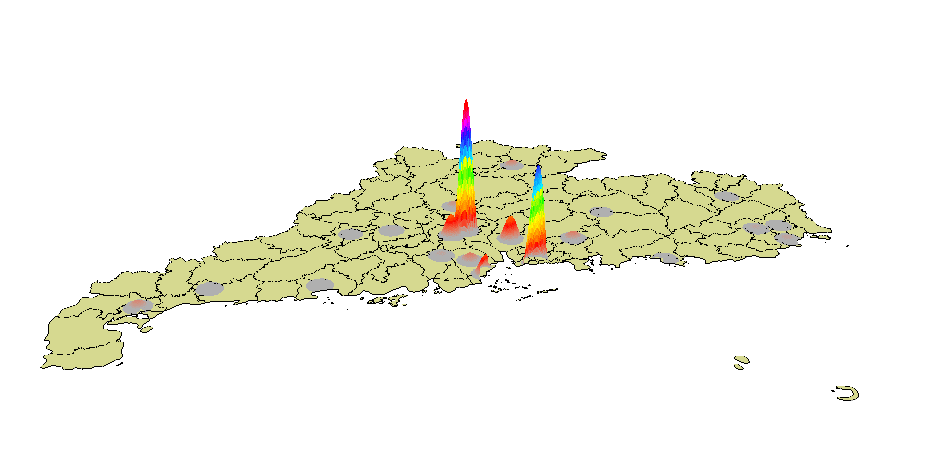


**Figure S15** Parameter setting for Guangdong in ArcScene

**S3.6 Hunan Province**


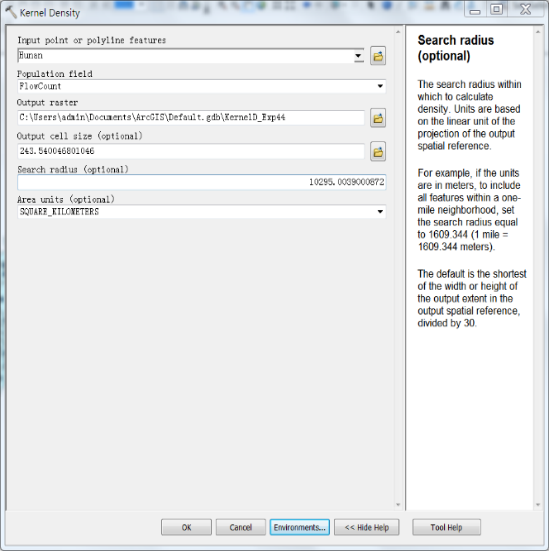

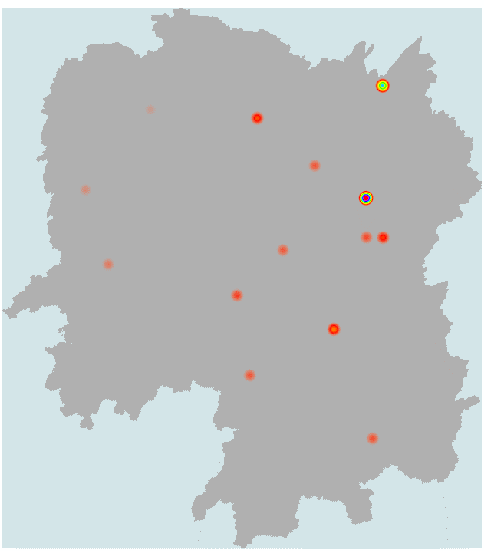


**Figure S16** Parameter setting for Hunan in ArcMap


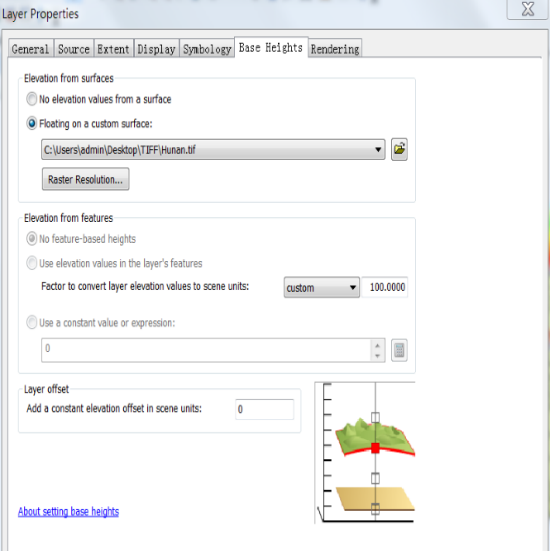

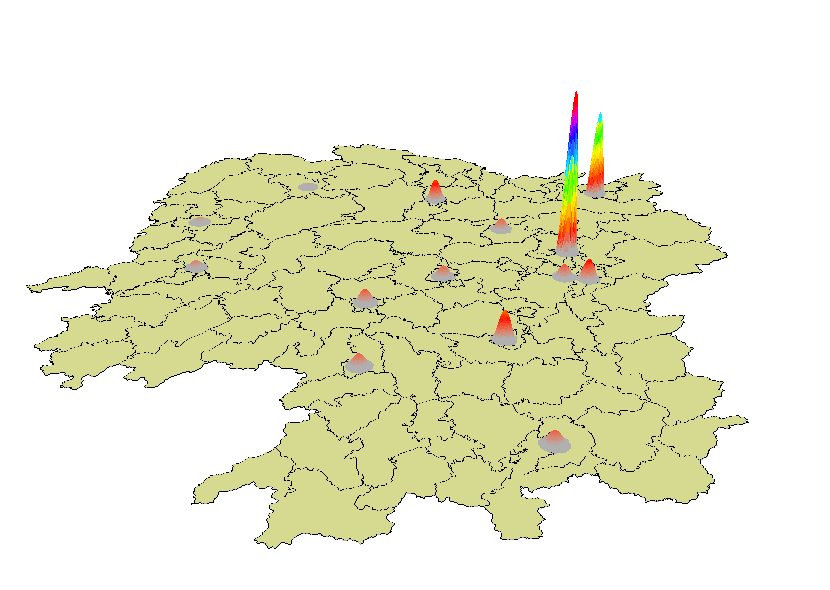


**Figure S17** Parameter setting for Hunan in ArcScene

**S3.7 Henan Province**


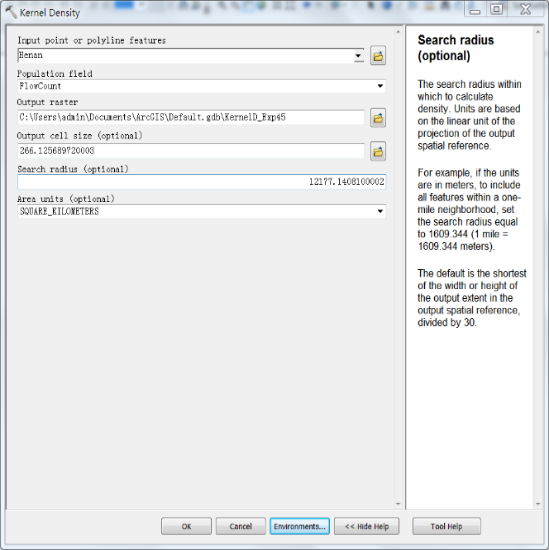

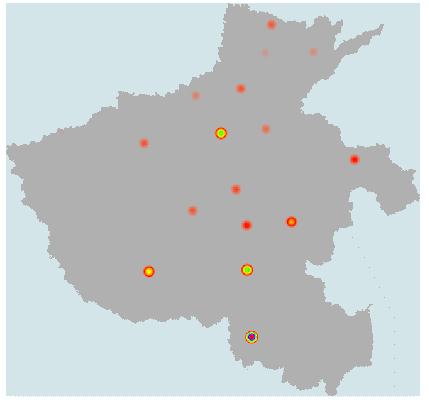


**Figure S18** Parameter setting for Henan in ArcMap


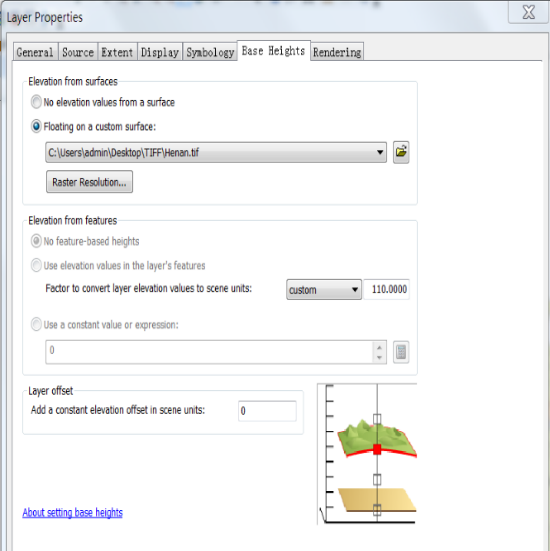

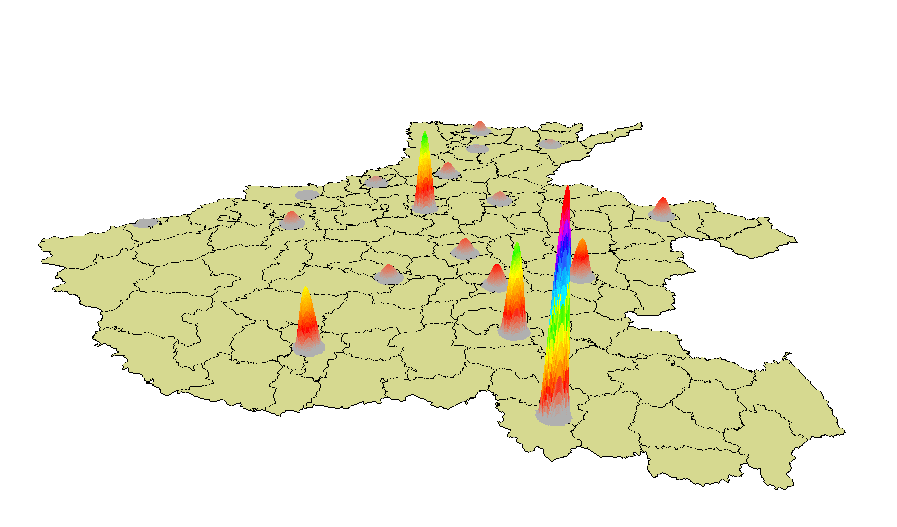


**Figure S19** Parameter setting for Henan in ArcScene

**S4. Parameter Setting in Gephi**


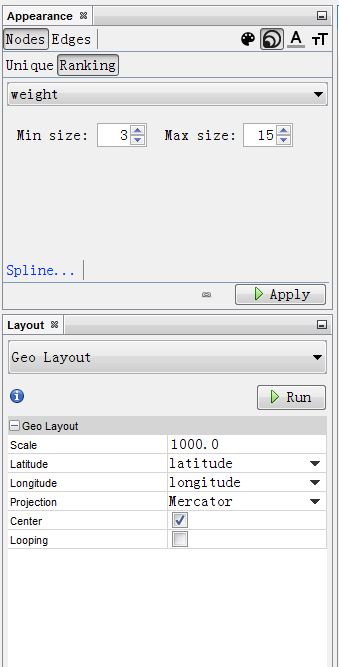

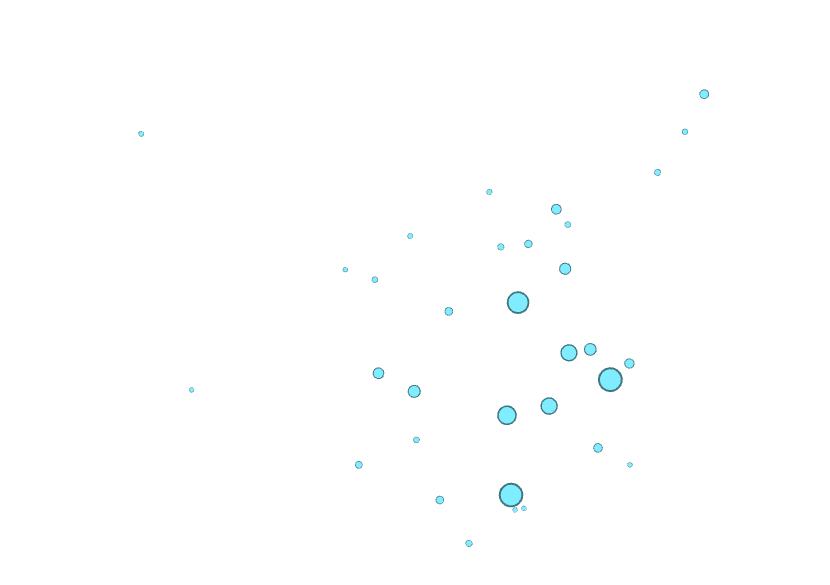


**Figure S20** Parameter setting for node size and layout in Gephi

1. Ministry of Industry and Information Technology of People’s Republic of China. http://www.miit.gov.cn [↑](#footnote-ref-1)
2. National Bureau of Statistics of China. http://www.stats.gov.cn [↑](#footnote-ref-2)
3. https://qianxi.baidu.com/ [↑](#footnote-ref-3)
